# An overview of research on air and environmental contamination with severe acute respiratory coronavirus virus 2 (SARS-CoV-2) in healthcare settings

**DOI:** 10.1017/ice.2020.1366

**Published:** 2020-12-10

**Authors:** Hajime Kanamori

**Affiliations:** Department of Infectious Diseases, Internal Medicine, Tohoku University Graduate School of Medicine, Sendai, Miyagi, Japan

*To the Editor—*I read with great interest a recent article by Cheng et al^1^ that examined the contamination status of air and environmental surfaces with severe acute respiratory syndrome coronavirus 2 (SARS-CoV-2) in airborne infection isolation rooms occupied by single symptomatic and asymptomatic patients with coronavirus disease 2019 (COVID-19). In this study, SARS-CoV-2 RNA was not detected in air samples and was infrequently detected in environmental samples (eg, patients’ mobile phones, bedrail, and toilet door handle) taken before daily cleaning and disinfection of the isolation rooms. Cheng et al^1^ concluded that rigorous hand hygiene, environmental cleaning and disinfection, and appropriate use of surgical masks were sufficient for healthcare infection prevention and control, except during aerosol-generating procedures. Other studies in different countries also investigated both air and environmental contamination with this novel coronavirus in healthcare settings, with variable contamination status findings (Table 1).^1–12^

**Table 1.** Air and Environmental Contamination With Severe Acute Respiratory Syndrome Coronavirus 2 in Healthcare Settings

Author, Year, Country	Air Sampling Situation	Patient Status	AGP Type	Air Sampling Method	Detection Method	Air Contamination	Result for Air/Environmental Samples
Cheng et al, 2020, China^1^	Airborne infection isolation rooms with 12 air changes/h; an air shelter (an umbrella fitted with a transparent plastic curtain) used to cover patients	Symptomatic and asymptomatic cases	No AGP	Air samples collected 10 cm from a patient’s chin with or without a surgical mask inside the air shelter using the Sartorius MD8 airscan sampler at a rate of 50 L/min for 20 min (1,000 L of air)	RT-PCR	All of the air samples negative (0/6); air outlet fan contamination 2/3	Negative/Positive
Chia et al, 2020, Singapore^2^	Airborne isolation rooms with 12 air changes/h and exhaust flow of 579.6 m^3^/h throughout the 4-h sampling period	Symptomatic and asymptomatic cases	No AGP	3 size fractions collected with NIOSH BC 251 bioaerosol samplers connected to SKC AirChek TOUCH Pumps or SKC universal air sampling pumps with 5,040 L of air from each patient’s room	RT-PCR	Air positive rate 66.7% (2/3); positive for particle sizes > 4 μm and 1–4 μm in diameter; negative for particle sizes < 1 μm; air exhaust vent positive rate 60% (3/5)	Positive/Positive
Guo et al, 2020, China^3^	Isolation ward of the ICU (12 air supplies and 16 air discharges/h) and general ward (8 air supplies/h, 12 air discharges/h); indoor air and air outlets sampled to detect aerosol exposure	Severe cases in ICU and mild cases in general ward	NA	SASS 2300 Wetted Wall Cyclone Sampler at 300 L/min for 30 min	RT-PCR	Air positive rate 35% (14/40) in ICU and 12.5% (2/16) in general ward; air outlet positive rate 66.7% (8/12) in ICU and 8.3% (1/12) in general ward	Positive/Positive
Jerry et al, 2020, Ireland^4^	Rooms of COVID-19 patients (intubated or receiving noninvasive ventilation)	Symptomatic cases	AGP	Air sampler SAS Super ISO 100	RT-PCR	All air samples negative (0/16: 8 from patient rooms and 8 from corridors of COVID-19 wards)	Negative/Positive
Lei et al, 2020, China^5^	ICU and isolation ward. The airflow in ICU rooms was a class-100 000 clean room with laminar flow. Average air change was 240/h to 360/h between 8 a.m. and 12 p.m.	Severe and critical cases	AGP; mechanical ventilation, bronchoscopy, intubation	NIOSH cyclonic bioaerosol sampler (for 4 hours at a flow rate of 3.5 L/min into 3 size fractions) and DingBlue sampler (at a flow rate of 14 L/min for 30 min); samplers placed on opposite sides at head of bed within 1 m of patient’s head at a height of 1.3 m	RT-PCR	2 air samples in bathroom on 2 different days positive	Positive/Positive
Li et al, 2020, China^6^	After 4 daily air disinfection by a plasma air sterilizer, aerosol samples were collected from various areas of a designated hospital for severe COVID-19 patients, including ICU ward with 12 air supplies/h and 16 discharges/h and isolation room with 8 air supplies/h and 12 discharges/h	Severe and critical cases	NA	Impingement air sampler BIO-Capturer-6 placed 1–1.5 m above floor in wards and 1–5 m from patients’ beds collected a total of 2,400 L air at a rate of 80 L/min for 30 min	RT-PCR	All aerosol samples negative (0/135)	Negative/Negative
Ong et al, 2020, Singapore^7^	Airborne infection isolation rooms (12 air exchanges/h)	Mid symptomatic cases	No AGP	SKC universal pumps for 4 h at 5 L/min in patient room and anteroom; collected on 2 days	RT-PCR	All air samples negative; air outlet fan contamination 2/3	Negative/Positive
Razzini et al, 2020, Italy^8^	COVID-19 ward with negative airflow system; air samples collected from 3 zones (contaminated, semicontaminated, and clean areas)	Patients intubated and supported by a respirator, and a patient not intubated and without CPAP nasal mask support	AGP; intubated, supported by a respirator, or not intubated and without CPAP nasal mask support	MD8 air sampler	RT-PCR	All air samples from contaminated area of ICU and corridor positive; samples from semicontaminated or clean areas negative	Positive/Positive
Santarpia et al, 2020 USA^9^	Negative pressure patient rooms with 12–15 air exchanges/h and hallways in the National Quarantine Unit on days 5–9 of occupancy and in the Nebraska Biocontainment Unit on day 10. Not all patients removed masks during air sampling; 58% of patients were symptomatic (eg, cough).	Mild symptomatic cases	No AGP	Sartorius Airport MD8 air sampler placed on bedside tables and nightstands at least 1 m away from patient; collected high-volume (50 L/min) stationary air samples. Personal Button Samplers (SKC) and AirChek pumps (SKC) used by study personnel to collect low-volume (4 L/min) personal air samples on 2 d during sampling activities	RT-PCR, viral culture	In-room air positive rate 63.2%; no virus cultured but viral proteins observed by immunofluorescence in hallway sample after 3 d cell culture	Positive/Positive
Wei et al, 2020, China^10^	Negative-pressure, non-ICU in a designated isolation ward between 10:30 a.m. and 1:00 p.m. during routine medical activities.	Asymptomatic and mild symptomatic cases	No AGP	Air sampler FSC-1V placed ∼0.6 m away from each patient and 1 m above floor in each room for 15 minutes at 100 L/min.	RT-PCR	All air samples negative; air exhaust outlets 50% positive (3/6)	Negative/Positive
Zhou et al, 2020, UK^11^	For the first procedure, before and during the procedure; for the other procedures, during the procedure only	Severe cases	AGP; tracheostomy	Coriolis μ air sampler to collect 1-m^3^ air samples	RT-PCR, viral culture	Air positive rate 6.4% (2/31) but no virus cultured; in operating theatres, 1/3 air samples collected during 3 tracheostomy procedures positive	Positive/Positive

Note. AGP, aerosol-generating procedure; COVID-19, coronavirus disease 2019; CPAP, continuous positive airway pressure; ICU, intensive care unit; NA, not applicable; National Institute of Occupational Safety and Health, NIOSH; PPE, personal protective equipment; RT-PCR, reverse transcription polymerase chain reaction; SARS-CoV-2, severe acute respiratory syndrome coronavirus 2.

These studies assessed air and environmental contamination with SARS-CoV-2 by either reverse-transcription polymerase chain reaction (RT-PCR) or viral culture or both. Detecting SARS-CoV-2 RNA in air or aerosol samples does not verify the presence of viable virus. Furthermore, methods of air sampling for data collection, analysis, and interpretation, including air sampler type, particle size, air volume, airflow rate, and sampling time and place are not standardized.^13^ Zhou et al^11^ reported that viral culture did not show viable SARS-CoV-2, even though 2 of 31 air samples (6.4%) and 23 of 218 surface samples (10.6%) were positive for its RNA (cycle threshold [Ct] value > 30 in all samples). In an experimental study, they allowed various dilutions of SARS-CoV-2 to dry on steel or plastic surfaces and found culturable SARS-CoV-2 in dried inoculum (Ct value < 30). After 3 days of culture, Santarpia et al^9^ observed viral proteins by immunofluorescence in a hallway sample, although they did not confirm cultivation of SARS-CoV-2.

Studies of air and environmental surfaces found that if air samples were positive for SARS-CoV-2 RNA, environmental surface samples were also (Table 1).^2,3,5,8,9,11^ Air samples taken <1 m from a patient receiving high-flow nasal cannula oxygen therapy were contaminated, but air and surface contamination levels were lower in intensive care units probably because of the use of closed-circuit ventilation systems.^11^ Several studies have shown that even when air samples were negative for SARS-CoV-2 RNA, environmental samples from air outlets were positive.^1,7,10^ Wei et al^10^ reported that surfaces in patient rooms with air exhaust outlets that tested positive for SARS-CoV-2 RNA were frequently contaminated (26.7%–95.7%), suggesting that small virus-laden particles are present around patients. In one study, environmental samples collected after cleaning and disinfection, and all air samples except for those from air exhaust outlets, were negative in RT-PCR, although the small volumes of the samples may have affected these results.^7^

Person-to-person SARS-CoV-2 transmission occurs primarily via respiratory droplets and contact, but some scientists suggested that airborne transmission (microdroplets or aerosols) also occurs.^14^ Several studies have shown that air samples were positive for SARS-CoV-2 RNA in isolation rooms with 12 air changes per hour.^2,3,9^ Viral RNA was detected in the air within 4 m of a patient, indicating possible aerosol transmission of SARS-CoV-2.^3^ Samples from the air around severely ill patients treated with aerosol-generating procedures were likely to be positive for SARS-CoV-2 RNA.^5,8,11^ However, the positivity rates of air samples collected around patients who did not receive aerosol-generating procedures have been discordant,^1,2,7,9,10^ while environmental surface samples were positive for SARS-CoV-2 RNA in all of these studies. Tang et al^15^ also reviewed the scientific evidence for aerosol transmission of SARS-CoV-2 and potential control measures in various situations and populations, highlighting that healthcare personnel are at high risk for aerosol transmission of SARS-CoV-2 in the closed hospital environment.^15^ Viral aerosol particles can be generated by mildly ill patients without a cough, leading to extensive environmental and potential aerosol contamination with SARS-CoV-2; however, no cases of COVID-19 were documented in healthcare personnel who took airborne precautions.^9^ On this basis, healthcare personnel should implement airborne precautions when performing aerosol-generating procedures in patients with COVID-19.
